# Changes in the epidemic patterns of respiratory pathogens of children in guangzhou, China during the COVID-19 pandemic

**DOI:** 10.1186/s12879-025-11215-8

**Published:** 2025-07-01

**Authors:** De-Feng Liang, Wen-Lin Guo, Dan-Ping Zhu, Su-Yun Li, Wei-Dong Zhu, Ying Li, Li Huang, Jun Shen, Pei-Qing Li

**Affiliations:** 1 Department of Emergency, Guangdong Provincial Clinical Research Center for Child Health, Guangzhou Women and Children’s Medical Center, Guangzhou Medical University, Guangzhou, Guangdong 510623 China; 2 Department of Hematology and Oncology, Guangdong Provincial Clinical Research Center for Child Health, Guangzhou Women and Children’s Medical Center, Guangzhou Medical University, Guangzhou, Guangdong 510623 China; 3 Department of Gastroenterology, Guangdong Provincial Clinical Research Center for Child Health, Guangzhou Women and Children’s Medical Center, Guangzhou Medical University, Guangzhou, Guangdong 510623 China

**Keywords:** Children, Respiratory pathogens, COVID-19, Non-Pharmaceutical interventions, Epidemiological characteristics

## Abstract

**Objective:**

To investigate the epidemiological characteristics of pediatric respiratory pathogens in Guangzhou, China, from 2018 to 2023 in the context of the COVID-19 Pandemic and to evaluate the impact of non-pharmaceutical interventions (NPIs) on the transmission dynamics and seasonal patterns of respiratory pathogens.

**Methods:**

A retrospective analysis was conducted at Guangzhou Women and Children’s Medical Center between January 2018 and December 2023. Pediatric patients who underwent the respiratory pathogens tests were enrolled in the study and divided into four groups by age: Infant group, Toddler group, Preschool group, and School-age group. The nasopharyngeal swab or bronchoalveolar lavage fluid (BALF) samples were collected for real-time fluorescence quantitative polymerase chain reaction (qPCR) test of respiratory pathogens. Ten common respiratory pathogens, including respiratory syncytial virus (RSV), Mycoplasma pneumoniae (MP) and Influenza A virus (FluA), were detected. In addition, the study period was divided into three phases: Pre-COVID-19 (2018–2019), COVID-19 (2020–2022), and Post-COVID-19 (2023). Detection rates, distribution patterns, and seasonal variations of respiratory pathogens were analyzed between different phases and different age groups.

**Results:**

This study included 317,828 pediatric patients (median age: 3.4 years, IQR: 1.3-6.0), from whom 1,160,764 respiratory pathogen tests were conducted. The overall pathogen detection rate was 8.02% (93,108/1,160,764). The positive rate during the COVID-19 phase (18.44%, 95% CI 14.89-22.00%) was significantly lower than that of the Pre-COVID-19 (27.55%, 95% CI 23.95–31.16%) and Post-COVID-19 (27.15, 95% CI 21.47–32.84%) phases (*P* < 0.001). No significant difference was observed between Pre-COVID-19 and Post-COVID-19 phases (*P* = 0.948). Different pathogens exhibited varying levels of suppression and recovery patterns, with some demonstrating altered seasonal patterns. The primary affected age groups shifted from infants and toddlers during the Pre-COVID-19 and COVID-19 phases to preschool and school-age children in the Post-COVID-19 phase. Co-infections were identified in 2,515 cases, with the highest rate observed during the Pre-COVID-19 phase (3.40%), followed by the COVID-19 (2.26%) and Post-COVID-19 (2.46%) phases.

**Conclusions:**

NPIs implemented during the early COVID-19 pandemic effectively suppressed the transmission of respiratory pathogens and disrupted their seasonal patterns. However, some pathogens gradually resumed activity during the mid-to-late COVID-19 phase, leading to atypical outbreaks. Following NPI relaxation, multiple pathogens rebounded during the Post-COVID-19 phase, posing significant challenges for the healthcare system. These findings offer valuable insights for guiding public health strategies and optimizing the prevention and control of respiratory infections.

**Clinical trial number:**

Not applicable.

## Introduction

Despite advancements in medical technology and improved hygiene conditions, acute respiratory infections (ARIs) remain a leading contributor to the global disease burden. ARIs account for a substantial proportion of pediatric outpatient visits, emergency department consultations, and hospital admissions, particularly in low- and middle-income countries [1, 2]. Respiratory pathogens demonstrate distinct seasonal distribution patterns and age-specific characteristics and are the primary cause of morbidity and mortality among children, particularly those under five years old, during the winter and spring seasons [1, 3]. These factors pose significant challenges to healthcare systems worldwide.

In December 2019, an outbreak of coronavirus disease 2019 (COVID-19), caused by severe acute respiratory syndrome coronavirus 2 (SARS-CoV-2), was first identified in Wuhan, Hubei Province, China. To mitigate the threat from this emerging infectious disease, China implemented a series of non-pharmaceutical interventions (NPIs) over three years. These included: (1) personal protective measures (e.g., mandatory mask-wearing); (2) social distancing measures (e.g., maintaining physical distance, restricting mobility, and limiting gatherings); and (3) quarantine and control measures (e.g., isolating confirmed cases and close contacts, enforcing strict screening and quarantine for inbound travelers) [4, 5]. These interventions not only significantly altered children’s social behavior patterns but also influenced the epidemiological characteristics of respiratory pathogens. Several studies have documented a substantial reduction in the detection rates of common respiratory pathogens, such as respiratory syncytial virus (RSV), Mycoplasma pneumoniae (MP), and influenza viruses, during the NPI implementation period [6–11].

Prolonged and stringent NPIs likely had a significant influence on the epidemiological patterns of respiratory pathogens. These insights are critical for improving future epidemic prevention and control strategies. However, most existing studies, both domestic and international, have primarily focused on individual pathogens or specific time periods. Consequently, there is a lack of comprehensive analyses addressing the long-term dynamics of multiple respiratory pathogens [12–14].

This study retrospectively analyzed respiratory pathogen detection data from January 2018 to December 2023 at Guangzhou Women and Children’s Medical Center, a major regional pediatric healthcare centera. The primary objective was to systematically assess the long-term impact of NPIs on the epidemiological patterns of multiple respiratory pathogens, thereby providing crucial evidence for optimizing public health strategies and enhancing the understanding of respiratory pathogen transmission dynamics.

## Methods

### Study design

This study was conducted at Guangzhou Women and Children’s Medical Center, recognized as the National Regional Medical Center for Children in South China. As the largest pediatric specialized medical institution in the region, the center managed over 4.5 million outpatient and emergency visits, as well as nearly 150,000 inpatient cases in 2023, highlighting its strong regional representativeness.

The inclusion criteria for this study were as follows: (1) Pediatric patients who underwent respiratory pathogen testing at the center between January 1, 2018, and December 31, 2023; (2) Age < 18 years; (3) Only the first respiratory pathogen test result was included if the patient had repeated tests of the same pathogen within 14 days. Patients were classified into four groups: Infant group (< 1 year), Toddler group (≥ 1 to < 4 years), Preschool group (≥ 4 to < 6 years), and School-age group (≥ 6 to < 18 years). The study period was categorized into three phases based on the timeline of major NPIs implemented in China during the COVID-19 pandemic. The Pre-COVID-19 phase (January 2018-December 2019) represented the period before the pandemic. The COVID-19 phase (January 2020-December 2022) covered the period of widespread implementation of stringent NPIs, including the ‘zero-COVID’ policy. The Post-COVID-19 phase (January 2023-December 2023) corresponded to the period after significant relaxation of these NPIs. This categorization reflects distinct public health environments influencing pathogen transmission. Respiratory pathogen testing was determined by the pediatrician based on the patient’s clinical conditions, targeting single or multiple pathogens. The following ten respiratory pathogens were tested: RSV, MP, Human adenovirus (HAdV), Influenza A virus (FluA), Influenza B virus (FluB), Chlamydia pneumoniae (CP), Human parainfluenza virus (HPIV), Human bocavirus (HBoV), Human metapneumovirus (HMPV), and Human rhinovirus (HRV). The study protocol was approved by the Ethics Committee of Guangzhou Women and Children’s Medical Center (505A01).

### Sample collection and laboratory testing

Respiratory specimens, including nasopharyngeal swabs and bronchoalveolar lavage fluid, were collected by professionally trained clinicians following standard operating procedures. The specimens were transported to the laboratory at 4 °C. To ensure the integrity and quality of the test results, nucleic acid extraction and qPCR analysis were consistently performed within 24 h of sample collection. Nucleic acids were extracted using an automated nucleic acid extraction system (E-Five, Guangzhou Institute of Respiratory Health Biosafety Technology Co., Ltd.). Real-time quantitative polymerase chain reaction (qPCR) was conducted for pathogen detection. Specific nucleic acid detection kits (typically utilizing PCR fluorescence probe methodology) for each of the ten respiratory pathogens listed (RSV, MP, HAdV, FluA, FluB, CP, HPIV, HBoV, HMPV, and HRV) were used. These kits were consistently sourced from certified national suppliers: Guangdong Huaruian Biology Co., Ltd., Guangdong Hecin-scientific Health Tech Co., Ltd., and Guangzhou Daan Gene Co., Ltd. All tests were performed strictly following the respective manufacturers’ instructions. Positive and negative controls were included in each batch of tests, and all experimental procedures were conducted by trained and certified laboratory technicians.

### Data processing and statistical analysis

Data collation and statistical analysis were performed using R-Studio software (2024.09.0 Build 375, Posit, PBC). Non-normally distributed data were expressed as median and interquartile range (IQR). 95% confidence intervals were also calculated. For multiple groups, one-way analysis of variance for normally distributed data and Kruskal–Wallis test for non-normally distributed data were used. Further comparisons between the two groups were performed using Dunnett’s test with Bonferroni correction. A two-sided P-value < 0.01 was considered statistically significant.

## Results

### Basic characteristics

The demographic characteristics and overview of pathogen detection are listed in Table 1. A total of 317,828 pediatric patients were enrolled in this study. The median age of the study population was 3.4 (IQR, 1.3-6.0) years, with an increasing trend noted over the study period. The lowest number of pathogen detecting cases was recorded during the COVID-19 phase (25,818–37,893 cases annually), while the highest was observed in the Post-COVID-19 phase (111,036 cases). The lowest pathogen positive rate was observed in 2020 (17.91%), while rates in 2019 (32.04%), 2022 (30.64%), and 2023 (30.38%) were similar.

**Fig. 1:**
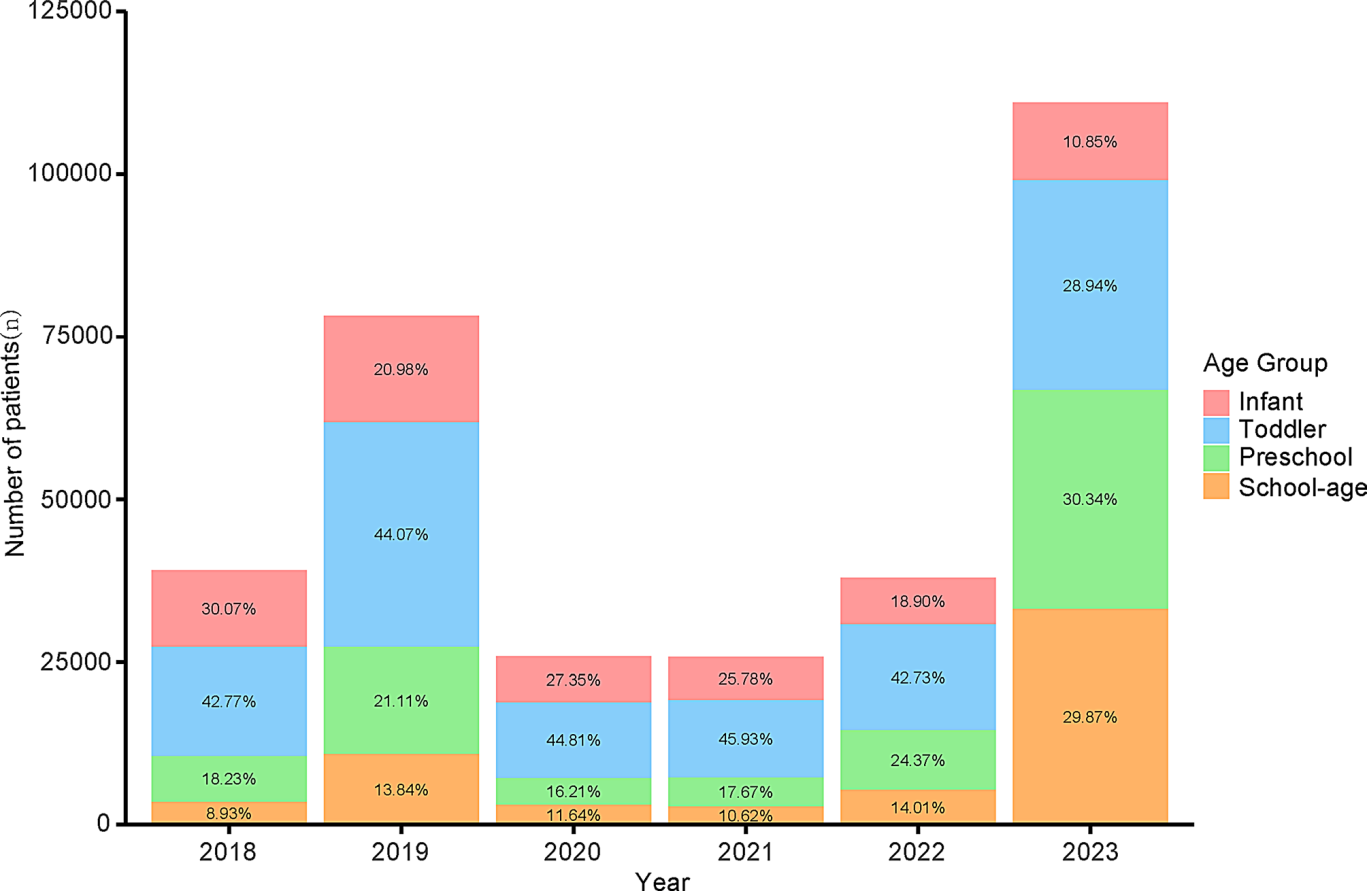
Annual Age Distribution of Pediatric Patients Undergoing Respiratory Pathogen Testing in 2018–2023. Stacked bar chart showing the annual number and proportion of enrolled patients in four defined age groups (Infant: <1 year; Toddler: ≥1 and < 4 years; Preschool: ≥4 and < 6 years; School-age: ≥6 and < 18 years)

In 2018, the Infant group accounted for 30.07% of all pathogen detecting cases, but the proportion steadily declined to 10.85% in 2023 (Fig. 1). The proportion of Toddler group represented the largest between 2018 and 2022 (42.77–45.93%) but decreased significantly to 28.94% in 2023. Conversely, the Preschool group (30.34%) and the School-age group (29.87%) accounted for the highest proportions in 2023.

**Table 1:** Demographic characteristics of patients and overview of pathogen detection

Year	Total	2018	2019	2020	2021	2022	2023
Total Patients	317,828	39,058	78,266	25,818	25,757	37,893	111,036
Median Age (Years)	3.4	1.9	2.8	2.4	2.5	3.3	5.1
IQR(Years)	1.3-6.0	08-3.4	1.2–3.9	0.9–4.3	0.9–4.3	1.3–5.2	2.6–7.5
Positive cases, n(%)	90,448 (28.46)	10,242 (26.22)	25,078 (32.04)	4,624 (17.91)	5,154 (20.01)	11,612 (30.64)	33,738 (30.38)
Total number of tests	1,160,764	152,666	248,511	110,393	150,355	179,039	319,800
Positive Pathogen number, n(%)	93,108 (8.02)	10,704 (7.01)	25,895 (10.42)	4709 (4.27)	5292 (3.52)	11,885 (6.64)	34,623 (10.83)

Positive Cases: the number of patients with at least one pathogen detection

Positive Pathogen number, n(%): The total count of all positive tests for any pathogen. A single patient with multiple pathogens detected (e.g., in a co-infection) contributes a count for each pathogen identified, while still being counted as a single ‘Positive case’

### Overall trends in pathogens

A total of 1,160,764 respiratory pathogen tests were conducted throughout the study period (Table 1), with an overall positive rate 8.02%. The lowest positive rate was observed during the COVID-19 phase (ranging from 3.52 to 6.64%), whereas the highest positive rate was recorded in the Post-COVID-19 phase (10.83%).

**Fig. 2:**
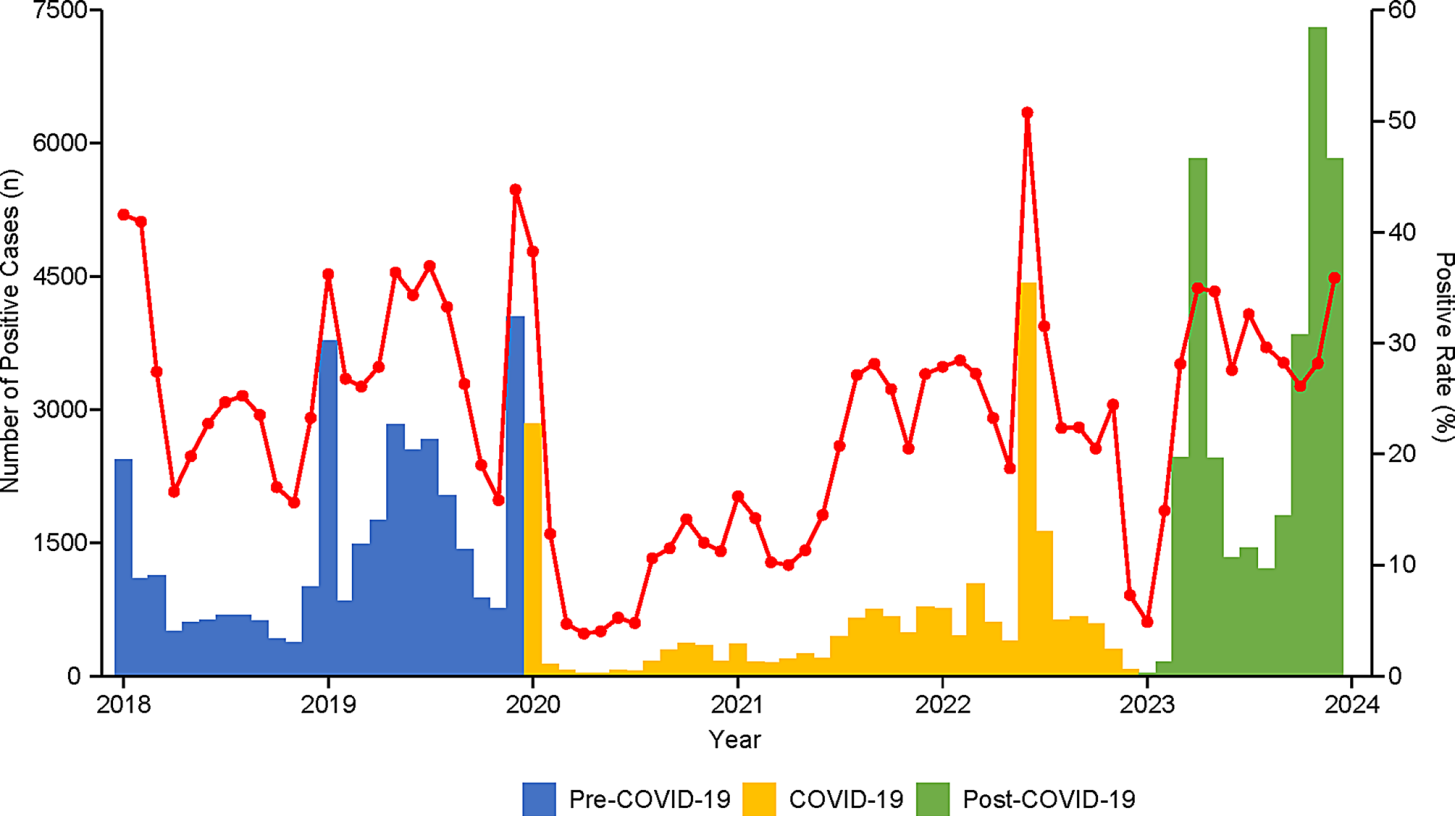
Monthly Trends of Overall Positive Respiratory Pathogen Cases and Detection Rates in 2018–2023. Bar chart (left Y-axis) shows monthly positive cases, color-coded by phase (Pre-COVID-19 (2018–2019), COVID-19 (2020–2022), Post-COVID-19 (2023) ); red line (right Y-axis) indicates monthly positive detection rate. Pathogen activity sharply decreased in early 2020, followed by suppressed/altered seasonality, and a marked resurgence in 2023

Monthly trends in the number of positive cases and the positive rate are illustrated in Fig. 2. During the Pre-COVID-19 phase, pathogen activity was predominant in the spring and winter seasons. In the early COVID-19 phase, both the number of positive cases and positive rates decreased to their lowest levels, followed by a gradual increase, which peaked in the summer of 2022. Notably, toward the end of the COVID-19 phase and the onset of the Post-COVID-19 phase, respiratory pathogen activity declined again to its lowest levels. Subsequently, the Post-COVID-19 phase began to exhibit pathogen activity patterns similar to those of the Pre-COVID-19 phase, with seasonal peaks that occurred in spring and winter.

The coefficient of variation (CV) during the COVID-19 phase was 56.90% (Table 2), notably higher than that of the Pre-COVID-19 phase (31.01%) and the Post-COVID-19 phase (32.95%). The pathogen positive rates showed significant differences among the three phases (χ² = 15.24, df = 2, *P* < 0.001). The positive rate during the COVID-19 phase (18.44%, 95%CI 14.89-22.00%) was significantly lower than that of the Pre-COVID-19 phase (27.55%, 95%CI 23.95–31.16%, Z = -3.2512, *P* = 0.002) and Post-COVID-19 phase (27.15, 95%CI 21.47–32.84%, Z = -3.0779, *P* = 0.003). No significant difference was observed between the Pre- and Post-COVID-19 phases (Z = 0.4786, *P* = 0.948).

**Table 2:** Summary of pathogen detection across different periods

Period	Month	Total Number	Positive Cases	Mean Monthly Positivity Rate (%)	95% CI(%)	CV(%)
Pre-COVID-19	24	117,324	35,320	27.55	23.95–31.16	31.01
COVID-19	36	89,468	21,390	18.44	14.89-22.00	56.90
Post-COVID-19	12	111,036	33,738	27.15	21.47–32.84	32.95

Pre-COVID-19: 2018–2019; COVID-19: 2020–2022: Post-COVID-19: 2023

CV(%): coefficient of variation, calculated as (standard deviation/mean × 100)

Mean Monthly Positivity Rate (%): Calculated as the average of the individual monthly positivity rates for the specified period

### Pathogen activity patterns

**Fig. 3:**
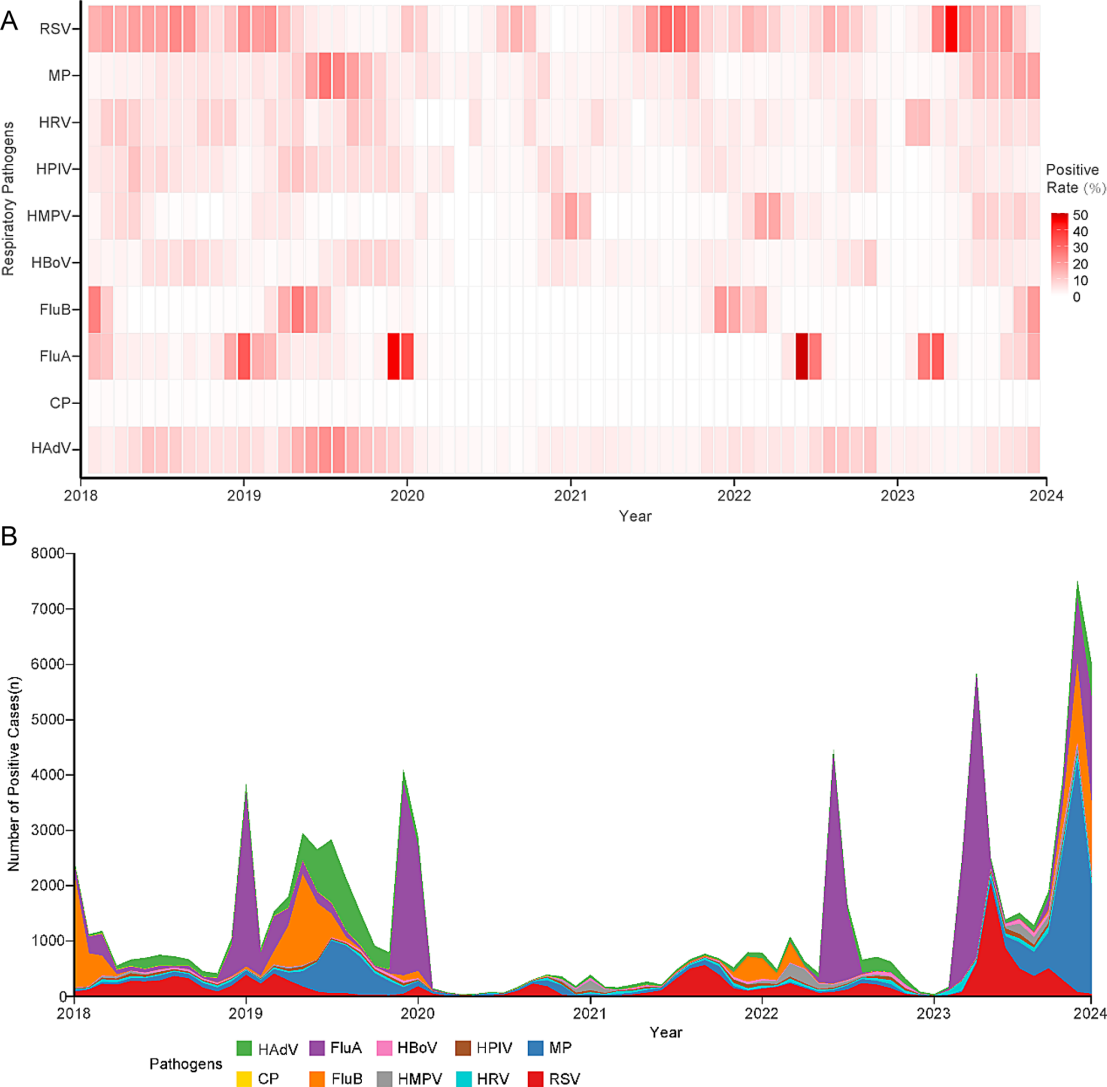
Monthly Trends in Detection Rates (**A**) and Positive Cases (**B**) for Ten Individual Respiratory Pathogens in 2018–2023. (**A**) Heatmap of monthly positive detection rates (%) for each pathogen. (**B**) Stacked area plot of monthly positive cases by pathogen, showing relative contributions. Most pathogens exhibited suppression during the COVID-19 phase, with notable resurgences for RSV, MP, FluA, and FluB in later periods. RSV: Respiratory syncytial virus; MP: Mycoplasma pneumoniae; HAdV: Human adenovirus; FluA: Influenza A virus; FluB: Influenza B virus; CP: Chlamydia pneumoniae; HPIV: Human parainfluenza virus; HBoV: Human bocavirus; HMPV: Human metapneumovirus; HRV: Human rhinovirus

The trends of positive cases and positive rates for the 10 pathogens across different phases are illustrated in Fig. 3. Pathogen activity declined during the early COVID-19 phase, recovered gradually in the later COVID-19 phase, reached a notable low point at the transition to the Post-COVID-19 phase (early 2023), and subsequently resurged. In the Post-COVID-19 phase, large-scale outbreaks of several pathogens, including RSV, MP, FluA, and FluB, were observed.

Among the pathogens, RSV was the first to recover after the initial suppression and experienced an off-season outbreak in the autumn of 2022. FluA and FluB demonstrated similar patterns, with minimal activity during the COVID-19 phase, followed by unusual outbreaks in the summer of 2022. MP exhibited low activity during the COVID-19 phase but showed the most significant rebound in the Post-COVID-19 phase. Notably, the rebound of MP occurred significantly later than that of RSV and influenza viruses (FluA and FluB). HMPV activity remained at consistently low levels throughout the study period, although a brief surge was observed during the COVID-19 phase.

### Age-Specific characteristics of infections

**Fig. 4:**
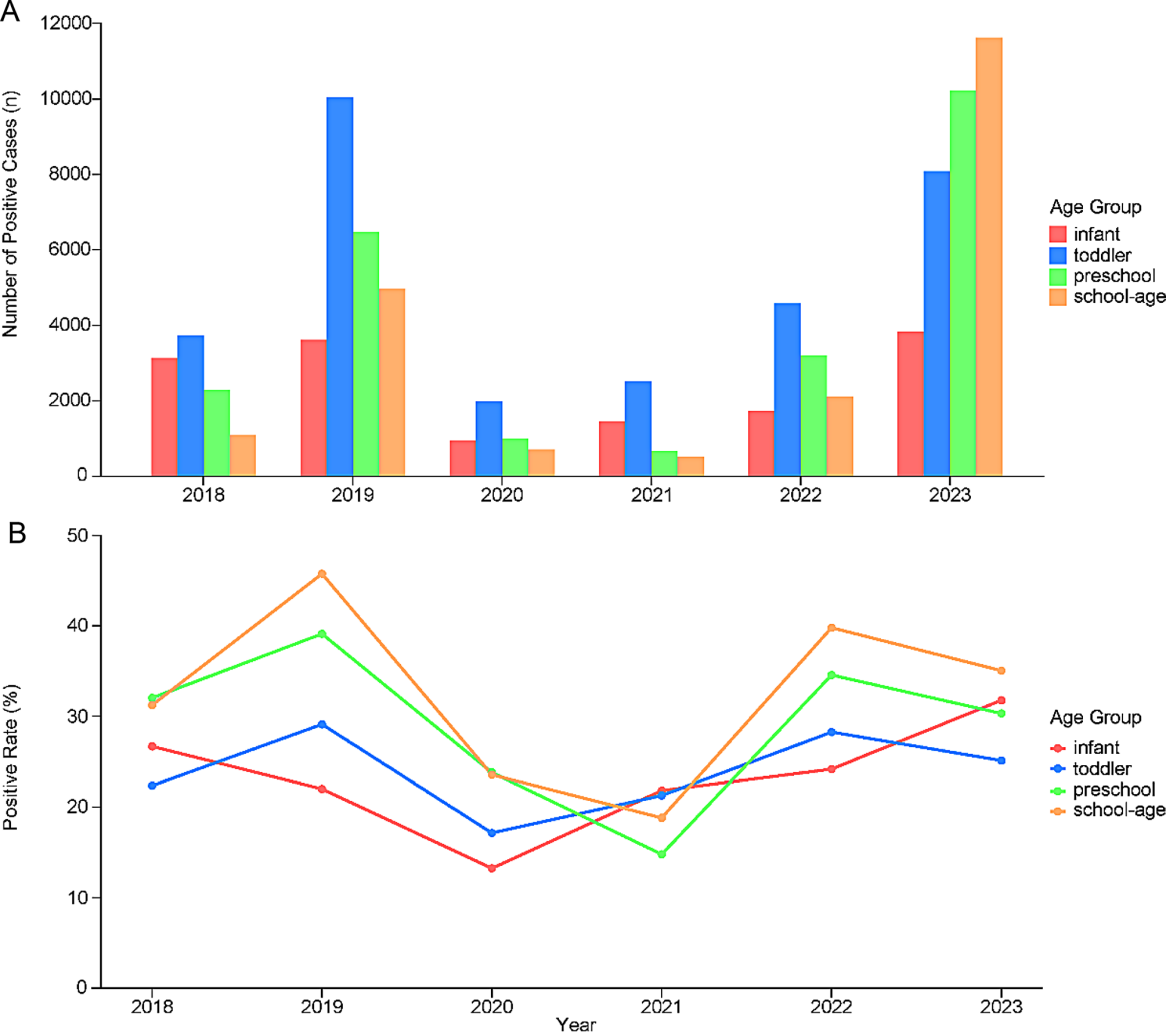
Annual Positive Respiratory Pathogen Cases (**A**) and Detection Rates (**B**) by Age Group in 2018–2023. Age groups are defined as: Infant (< 1 year), Toddler (≥ 1 to < 4 years), Preschool (≥ 4 to < 6 years), School-age (≥ 6 to < 18 years). (**A**) Bar chart of annual positive cases by age group. (**B**) Line graph of annual detection rates by age group (calculated as: Positive Cases in Age Group for that year / Total Enrolled Patients in Age Group for that year × 100%). While rates initially declined across all age groups, a rebound occurred with infections becoming most prevalent in preschool and school-age children in 2023

The infection characteristics differed among age groups, as shown in Fig. 4. During the early COVID-19 phase, pathogen infection rates declined across all age groups, reaching the lowest levels in the Infant group and the Toddler group in 2020, and in the Preschool group and the School-age group in 2021. Following these nadirs, infection rates in all age groups began to rise. During the Pre-COVID-19 and COVID-19 phases, respiratory pathogen infections were predominantly observed in the Infant and the Toddler groups. In contrast, during the Post-COVID-19 phase, infections were most prevalent in the Preschool and the School-age groups.

**Fig. 5:**
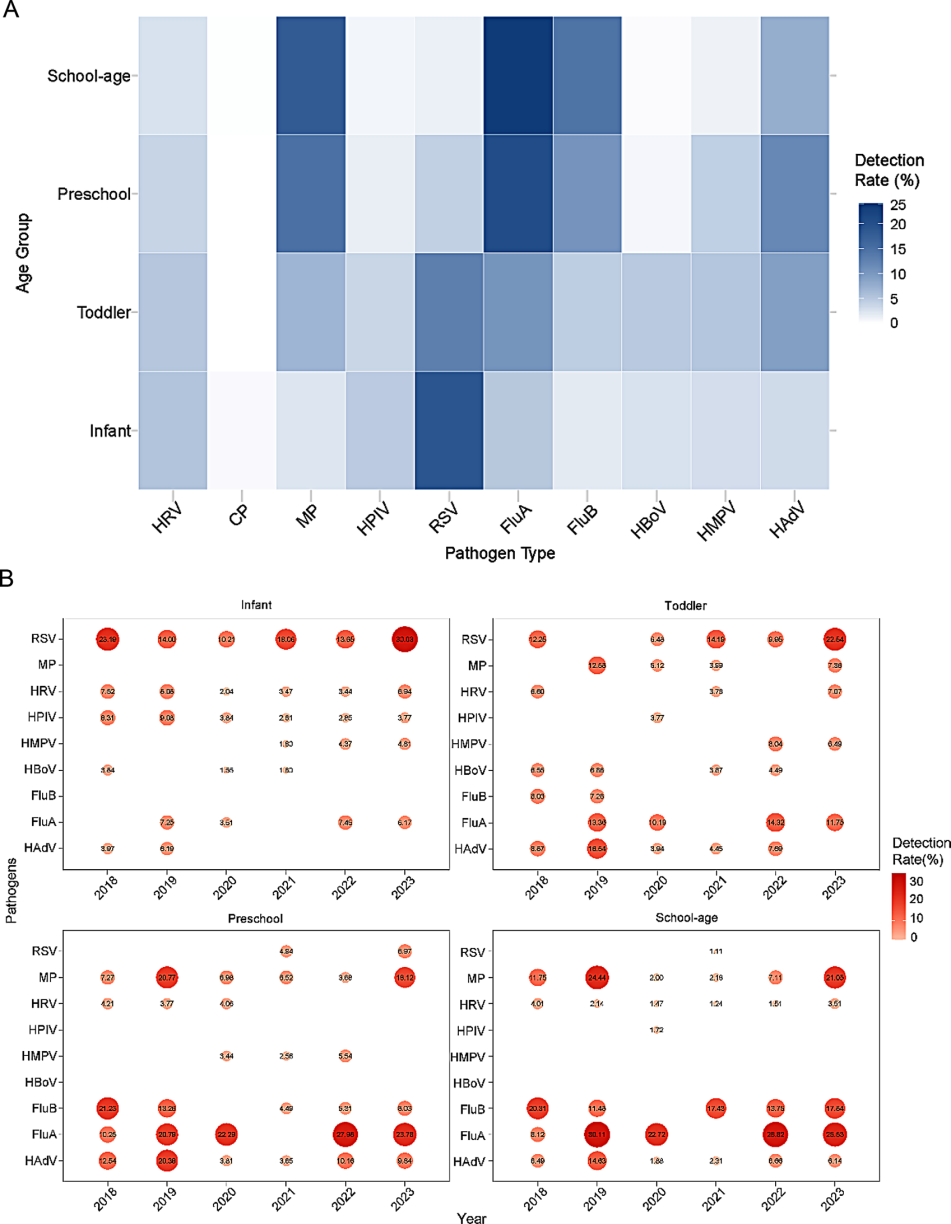
Annual Detection Rates of Specific Respiratory Pathogens by Age Group in 2018**–**2023. Age groups are defined as in Fig. 4. (**A**) Heatmap displaying annual detection rates (%) for ten respiratory pathogens by age group and year (darker color representing higher rates). (**B**) Bubble charts showing annual detection rates (%) for the top 5 predominant pathogens in each age group; bubble size and color intensity (e.g., darker red) indicate higher rates (empty spaces: pathogen not in top 5 for that age/year). Distinct age-related pathogen susceptibilities (e.g., RSV in infants; MP, FluA in older children) and temporal shifts in these patterns were evident

The susceptibility to different pathogens varied across age groups, as shown in Fig. 5A. For instance, RSV exhibited the highest infection rates in the Infant group, with a decreasing trend observed as age increased, indicating a negative correlation with age. Similarly, HRV and HPIV displayed comparable patterns. In contrast, FluA, FluB, and MP exhibited the highest infection rates in the School-age group, demonstrating a positive correlation with age. HMPV and HAdV predominantly affected the Toddler group and the Preschool group.

The top five predominant pathogens in each age group remained consistent throughout the study period, as shown in Fig. 5B. RSV consistently ranked as the most prevalent pathogen in the Infant group, while RSV, HAdV, and FluA were dominant in the Toddler group. In the Preschool and School-age groups, MP, FluB, and FluA were the most prevalent pathogens.

### Analysis of Co-infections

During the study period, 2,515 co-infection cases, defined as the simultaneous detection of two or more pathogens, were identified. The Toddler group accounted for the highest proportion (1,145 cases, 45.53%), followed by the Infant group (664 cases, 26.40%) (Table 3). The three most common pathogen combinations were MP + HAdV (15.51%), RSV + HAdV (7.63%), and MP + FluA (5.45%). The three most frequently detected pathogens in co-infections were HAdV (1,043, 41.47%), MP (1,004, 39.92%), and RSV (749, 29.78%).

The prevalence of co-infection was significantly higher during the Pre-COVID-19 phase (3.40%, 1,202/35,320) compared to the COVID-19 phase (2.26%, 483/21,390) and the Post-COVID-19 phase (2.46%, 830/33,738), and these differences were statistically significant (*P* < 0.001).

**Table 3:** Characteristics and patterns of Co-infections

Category	Subcategory	Cases(*n*)	Percentage/Frequency (%)
Age group	Infant	664	26.40
	Toddler	1145	45.53
	Preschool	419	16.67
	School-age	287	11.41
Co-infection Patterns	MP + HAdV	390	15.51
	RSV + HAdV	192	7.63
	MP + FluA	137	5.45
	RSV + MP	133	5.29
	RSV + HBOV	133	5.29
	MP + FluB	133	5.29
Pathogens	HAdV	1043	41.47
	MP	1004	39.92
	RSV	749	29.78
	HRV	542	21.55
	HBoV	493	19.60
	HPIV	395	15.70
	FluA	356	14.16
	FluB	291	11.57
	HMPV	264	10.50
	CP	38	1.51

Co-infection Patterns: the top 5 most frequent combinations of pathogens in co-infections, ranked by frequency (%)

Pathogens: the most frequently involved pathogens in Co-infections. Frequency (%) is calculated as: Frequency (%) = (Number of Cases Involving a Pathogen / Total Co-infection Cases) × 100%

## Discussion

This study systematically analyzed respiratory pathogen data from 317,828 pediatric patients in Guangzhou, China, spanning the years 2018–2023. The findings of this study highlighted significant changes in the epidemiological characteristics of respiratory pathogens across different phases, age-specific patterns of respiratory infection, and the prevalence of co-infections. The implementation of NPIs during the COVID-19 pandemic, as observed in this study, effectively suppressed respiratory pathogen transmission and disrupted their traditional seasonal patterns. However, during the Post-COVID-19 phase, the relaxation of NPIs led to a significant rebound of pathogen activity, a finding consistent with previous studies [15–19]. Notably, this study showed that long-term NPIs were insufficient to sustainably suppress pathogen transmission. During the mid-to-late COVID-19 phase, certain pathogens exhibited varying degrees of resurgence, with some reaching outbreak levels.

During the COVID-19 phase, overall pathogen positive rates significantly declined across all age groups, highlighting the broad suppressive effect of NPIs on respiratory pathogen transmission [20–22]. However, the impact of NPIs varied across different pathogens. For example, FluA and FluB nearly disappeared during the first two years of COVID-19 pandemic in our cohort. In contrast, RSV was the earliest pathogen to recover after maintaining a low activity level and exhibited an off-season outbreak in the autumn of 2022. A similar shift in RSV seasonal changes has been reported in other regions [23, 24]. Moreover, FluA exhibited an unusual off-season outbreak in the summer of 2022 in our findings. These findings suggest that long-term NPIs not only suppress pathogen activity but also alter their seasonal transmission patterns [25, 26].

Other pathogens, including MP, HRV, HMPV, HPIV and HAdV, exhibited low activity levels throughout the COVID-19 phase. This variability may be attributed to differences in biological characteristics and transmission pathways of different pathogens [27]. Toward the end of the COVID-19 phase and the onset of the Post-COVID-19 phase, respiratory pathogen activity dropped to its lowest levels again, possibly due to the disruptive effects of the SARS-CoV-2 pandemic temporarily suppressing the transmission of other respiratory pathogens [10, 28].

In the Post-COVID-19 phase, as SARS-CoV-2 prevalence declined, respiratory pathogens exhibited significant rebound outbreaks, with a trend of co-prevalence of multiple pathogens. This pattern of resurgence, including atypical peaks for viruses such as influenza and RSV following the widespread relaxation of NPIs, has been documented in various regions and is supported by meta-analytic data suggesting increased pathogen activity after interventions were relaxed [29]. This phenomenon, coupled with changes in pathogen seasonal patterns, can be attributed to the following mechanisms: first, pathogens may have evolved mechanisms to overcome the constraints imposed by NPIs, resulting in atypical transmission patterns. Second, public adherence to long-term NPIs, such as social distancing and personal protection, may have declined over time [25]. Reduced exposure to respiratory pathogens among children, particularly the infants born during the COVID-19 period, led to a widespread decline in herd immunity—a phenomenon termed “immunity debt” [30]. Herd immunity plays a critical role in shaping the seasonal transmission patterns of respiratory pathogens [31]. When NPIs were lifted, populations with weakened immunity became highly susceptible, leading to large-scale outbreaks [32–34]. Studies have demonstrated a positive correlation between the duration of NPIs and the magnitude of post-NPI pathogen rebound: the longer the NPIs were in place, the stronger the subsequent rebound [35, 36].

It is crucial to emphasize that the potential risk of “immunity debt” does not negate the value of NPIs. During the early stage of newly emerging pathogen, when knowledge of its transmission dynamics and pathogenicity is limited, NPIs remain the most effective strategy for mitigating pathogen transmission. Numerous studies have demonstrated the effectiveness of NPIs in breaking the transmission chain of respiratory pathogens [37].

Notably, MP exhibited a distinct pattern during the Post-COVID-19 phase compared to other pathogens. Its rebound was both delayed and more intense, with infection numbers significantly surpassing Pre-COVID-19 levels. Some studies have suggested that the pathogenicity of MP may have increased during this period [38]. Furthermore, the delayed re-emergence of MP is atypical and may be attributable to its unique biological characteristics [39].

The altered epidemiological characteristics of pathogens also resulted in changes to the age distribution of infected populations, a trend also noted in broader systematic analyses concerning specific pathogens like RSV [30]. During the COVID-19 phase, NPIs reduced infection rates across all age groups. However, the infant and toddler groups remained the primary affected populations of respiratory pathogens, consistent with Pre-COVID-19 patterns [40, 41]. In contrast, during the Post-COVID-19 phase, the Preschool and the School-age groups became the most affected populations for the first time. The observed shift can be attributed to the higher susceptibility of Preschool and School-age children to MP and influenza viruses, which were the predominant pathogens in the Post-COVID-19 phase [41]. Furthermore, the resumption of school and social activities in these age groups significantly increased their exposure risk to respiratory pathogens.

This study revealed that HAdV + MP was the most common co-infection combination, consistent with the findings of some studies [42, 43]. However, other studies have reported HRV and MP as the most frequent combination [44]. The co-infection rate declined significantly during the COVID-19 phase and partially recovered in the Post-COVID-19 phase, but it did not return to the Pre-COVID-19 levels. This trend may be associated with the predominance of infections in younger populations, suggesting that interactions between different pathogens remained relatively stable despite NPI interventions.

This study provides valuable reference data for understanding the epidemiology of respiratory pathogens and informing the development of prevention strategies. However, several limitations should be acknowledged. First, our inclusion of all pediatric patients undergoing pathogen testing without strict, predefined ARI diagnostic criteria means that a positive qPCR result may not always indicate the primary cause of acute illness (e.g., due to asymptomatic carriage or co-infection). Second, as a hospital-based, single-center study in Guangzhou, findings may not fully represent milder community infections, and generalizability to other regions is limited. Additionally, changes in health-seeking behavior during the pandemic could have influenced case detection. Finally, this observational study identified temporal associations; thus, causality (e.g., for NPI impacts) cannot be definitively established, and other unanalyzed factors (such as viral competition or pathogen evolution) may have contributed to the observed shifts.

## Conclusion

Our study reveals that NPIs during the COVID-19 pandemic profoundly reshaped pediatric respiratory infection epidemiology in Guangzhou (2018–2023). Initial suppression was followed by significant post-NPI pathogen rebounds and altered seasonality, with a notable shift in affected age groups towards older children, likely influenced by a temporary ‘immunity gap’. These findings underscore the critical need for enhanced surveillance systems to anticipate atypical outbreaks, proactive healthcare preparedness for unusual pathogen activity, and adaptive public health strategies, including optimized vaccination programs. Continued research into the mechanisms of these altered transmission dynamics and the long-term immunological impacts of NPIs on children is crucial.

## Data Availability

The datasets used and/or analysed during the current study are available from the corresponding author on reasonable request.

## Abbreviations

ARIs: Acute respiratory infections
NPIs: Non-pharmaceutical interventions
COVID-19: Coronavirus disease 2019
SARS-CoV-2: Severe acute respiratory syndrome coronavirus 2
RSV: Respiratory syncytial virus
MP: Mycoplasma pneumoniae
HAdV: Human adenovirus
FluA: Influenza A virus
FluB: Influenza B virus
CP: Chlamydia pneumoniae
HPIV: Human parainfluenza virus
HBoV: Human bocavirus
HMPV: Human metapneumovirus
HRV: Human rhinovirus
